# Interventions to reduce discrimination and stigma: the state of the art

**DOI:** 10.1007/s00127-017-1341-9

**Published:** 2017-01-31

**Authors:** Petra C. Gronholm, Claire Henderson, Tanya Deb, Graham Thornicroft

**Affiliations:** Health Services and Population Research Department P029, David Goldberg Centre, King’s College, London Institute of Psychiatry, Psychology and Neuroscience, De Crespigny Park, London, SE5 8AF UK

**Keywords:** Discrimination, Stigma, Mental disorders, Attitudes, Behaviour, Intergroup contact

## Abstract

**Background:**

There is a rich literature on the nature of mental health-related stigma and the processes by which it severely affects the life chances of people with mental health problems. However, applying this knowledge to deliver and evaluate interventions to reduce discrimination and stigma in a lasting way is a complex and long-term challenge.

**Methods:**

We conducted a narrative synthesis of systematic reviews published since 2012, and supplemented this with papers published subsequently as examples of more recent work.

**Results:**

There is evidence for small to moderate positive impacts of both mass media campaigns and interventions for target groups in terms of stigma-related knowledge, attitudes, and intended behaviour in terms of desire for contact. However, the limited evidence from longer follow-up times suggests that it is not clear whether short-term contact interventions have a lasting impact.

**Conclusions:**

The risk that short-term interventions may only have a short-term impact suggests a need to study longer term interventions and to use interim process and outcome data to improve interventions along the way. There is scope for more thorough application of intergroup contact theory whenever contact is used and of evidence-based teaching and assessment methods when skills training is used for target groups.

## Introduction

Mental health-related discrimination and stigma are global, multifaceted problems. Research and intervention in this field have applied a variety of definitions, targets and outcomes. An overview of these means of describing, clarifying and classifying the stigma concept is provided by, for example, Pescosolido and Martin [1]. They outlined two broad perspectives—experiential and action-orientated—through which stigma can be categorised.

The experiential perspective distinguishes between whether stigma is (1) perceived (a belief “most people” are considered to hold), (2) endorsed (expressing agreement with stereotypes/prejudice/discrimination), (3) anticipated (expecting an experience of prejudice/discrimination), (4) received (overt experiences of rejection or devaluation), or (5) enacted (exhibiting discriminatory behaviours).

The action-oriented view considers who (or what) gives or receives the stigma. From this perspective, distinctions are made between (1) public stigma (stereotypes, prejudice and discrimination as endorsed by the general population); (2) structural stigma (prejudice and discrimination through laws, policies, and constitutional practices); (3) courtesy stigma (stereotypes, prejudice and discrimination acquired through a connexion with a stigmatised group/person); (4) provider-based stigma (prejudice and discrimination by occupational groups designated to provide assistance to stigmatised groups), and; (5) self-stigma (when people who belong to a stigmatised group legitimise publicly held stereotypes and prejudice, and internalise these by applying them to themselves).

As expected for such a multifaceted phenomenon, the impacts of experienced and anticipated discrimination in combination are severe: poor access to mental [2, 3], and physical healthcare [4]; reduced life expectancy [5, 6]; exclusion from higher education [7, 8] and employment [9]; increased risk of contact with criminal justice systems; victimisation [10]; poverty and homelessness. For many people, these consequences have been described as worse than the experience of the mental illness itself [11]. While there is more than one theory of stigma, they share an understanding that it constitutes a significant public health concern [12, 13]. Some governments and non-governmental organisations have also recognised this. One of the six key objectives of the UK Government’s mental health strategy 2011–15 specified the need to ensure fewer people experience stigma and discrimination due to their mental illness [14]. The World Health Organisations’ Mental Health Action Plan 2013–2020 specifies that people affected by mental illness should be able to participate fully in society and at work, free from stigmatisation and discrimination [15].

## Anti-stigma interventions: the state of the art

In common with any public health intervention, programmes to reduce discrimination and stigma must be based on a series of decisions, in this case: the scope to mental disorders to be included, whether explicitly or implicitly; the level of intervention, whether structural, interpersonal, or self-stigma; whether to take a whole population approach versus choosing target groups, and if the latter, which groups are priority targets in terms of either the frequency and/or severity of the impact on people with mental health problems; what approach an intervention for a given group and at a given level should take; and how to evaluate the impact.

It is to be hoped that these decisions will be based on existing evidence and developmental work carried out by those delivering a programme. However, it seems likely that decision-making will also reflect who the decision-makers are. Traditionally, anti-stigma programmes were conducted by or with considerable involvement from groups representing psychiatric expertise such as the World Psychiatric Association and Royal College of Psychiatrists (regarding programmes in the UK). However, more recent programmes have greater leadership from advocacy groups and mental health charities. These groups have often identified mental health professionals as a source of stigmatisation and question whether they are therefore in a credible position to lead anti-stigma programmes [16]. Another potentially important influence on decision-making and credibility is the source of programme funding. For example, pharmaceutical company involvement may be perceived as a self-interested attempt to expand the market for psychotropic medicine as a result of increased help-seeking, on which stigma has a negative impact [2]. Government funding may be perceived as affecting the choice of targets or methods for a programme; this will be discussed later when we consider interventions to reduce structural discrimination.

Anti-stigma strategies have been categorised in terms of education (replacing myths about mental illness with accurate knowledge), contact (using direct or indirect—i.e. parasocial—interactions with people who have a mental illness to challenge prejudice), and protest (attempts to suppress stigmatising attitudes and representations of mental illness) [17]. Education and contact have been found to be the most commonly used. However, it should be noted that educational approaches vary widely in terms of what information they aim to convey. For example, mental health literacy programmes aim to increase knowledge of mental health problems, improve attitudes, and stimulate helping behaviours, [18–20], while rights-based programmes such as See Me in Scotland (https://www.seemescotland.org/our-movement-for-change/change-networks/human-rights/) focus on the legal rights of people with mental health problems.

Early anti-stigma efforts often used educational approaches. For example, work in the 1950s by Cumming and Cumming in a town in Canada [21] attempted to reduce stigma through providing mental health education via group discussions and films. Also in the UK, the ‘Defeat Depression’ campaign in the early 1990s aimed to reduce stigma through provision of information on depression for the public and professionals [22]. Interventions for health professionals often rely on educational approaches [23]. Over time, the use of intergroup contact has increased, especially following a meta-analysis by Corrigan and colleagues in 2012 which highlighted its effectiveness [24]. Below, we discuss how these approaches have been used both separately and together in population-level interventions and those targeted to specific groups.

## Anti-stigma interventions for the general public

At the time of writing, there has been a proliferation of national and regional programmes which have either recently finished or are ongoing. The summary below exemplifies the state of the art rather than providing a comprehensive description. ‘Beyondblue’ is a depression-specific programme in Australia which has been associated with improved public attitude and knowledge [25], with greater effects associated with greater exposure to the programme [26]. The ‘Like Minds, Like Mine’ anti-stigma programme in New Zealand resulted in reduced overall levels of discrimination [27], and there have also been indications of improved knowledge and attitudes following this work. ‘See Me’ is a national programme aiming to end mental health-related stigma and discrimination running in Scotland. When the findings of cross-sectional population surveys of public attitudes towards people with mental illness between 1994 and 2003 were compared between Scotland and England (where no comparable programme was underway), attitudes in England were significantly deteriorating whereas no comparable pattern was noted in Scotland where attitudes remained largely unchanged [28]. The ‘Hjärnkoll’ programme in Sweden started in 2010; by 2014, its contact-based strategy had achieved a positive impact on mental health literacy, attitudes and intended social contact with people with mental illness [29].

The ‘Time to Change’ programme in England (launched in 2008) and ‘Opening Minds’ in Canada (launched in 2009) [30] allow comparison of two programmes adhering to different frameworks. Whereas the Time to Change programme used a public health perspective, defining stigma in terms of problems relating to knowledge, attitudes and behaviours [11, 31], the Opening Minds programme used a sociological framework where stigma is considered reflective of the co-occurrence of labelling, stereotyping, separation, status loss and discrimination [32, 33]. Both programmes were built on evidence-based approaches to stigma reduction, with an emphasis on contact-based education strategies. There were, however, differences in how these strategies were delivered. Time to Change primarily aimed to target the general population via large-scale mass media social marketing campaigning. The initial focus was on education-based “myth busting”, followed by a focus on reducing prejudice and changing behaviours. Additionally, Time to Change involved local initiatives and work with target groups such as medical students and employers [34, 35]. In contrast, Opening Minds did not include a mass media element, after a short-term media campaign suggested little or no impact on stigma-related knowledge or desire for social distance [36]. Rather, it focused solely on intensive, targeted work with specific target groups across the country—young people, healthcare providers, the news media and the workforce—through grassroots input and community programmes [36].

Systematic academic evaluation has been carried out on several programme components of both Time to Change and Opening Minds, and on the overall impact of Time to Change among the general population and among users of mental health services [30]. The results of these evaluations indicate that both programmes have been successful in reducing stigma. In England, following Time to Change, benefits were observed in terms of population-level improvements in stigma-related knowledge, attitudes, social distance and reported contact with people having mental illness, albeit some of these effects emerged slowly [37, 38]. Positive changes were also evident in mental health service users’ reduced reporting of experiences of discrimination [39]. Population-level changes were not achievable in Canada as the programme was conducted on a local level. Evaluation of these local efforts did, however, indicate positive changes. For example, interventions focusing on high school students were generally successful in improving students’ intended behaviour towards people with a mental illness [40], and similarly, programmes amongst healthcare providers generally produced positive results in terms of their attitudes [41]. The goal within Opening Minds is to replicate successful programmes nationally; this work currently involves a national scale-up underway with the efforts focusing on young people.

Regarding identifying mechanisms of successful stigma change, albeit Time to Change included smaller efforts with a local or target focus, the intended population-level exposure to the mass media elements of the programme makes it impossible to examine the impact of these components separately. In contrast, Opening Minds built on community-based efforts only, which were all based on contact-based education strategies but were heterogeneous in nature [30]. This variability has enabled identification of how to effectively tailor stigma interventions for different populations [41], and what the effective programme ingredients are [42]. These findings are discussed further below in relation to work focused on key target groups.

## Anti-stigma interventions for key target groups

Stigma reduction within specific groups can in theory advance life opportunities for people with mental illness [11, 43]. Target groups have been identified on the basis of: high levels of contact with service users (healthcare professionals), position of power (law enforcement officers), or potential for changing the future (students and young people) [43].

### Healthcare professionals

Evidence regarding anti-stigma efforts amongst healthcare professionals has been discussed, for example, by Henderson and colleagues [23]. Their review identified 16 intervention studies examining stigma reduction in relation to mental health generally or specific mental health conditions (e.g. borderline personality disorder, substance misuse) amongst various groups of healthcare and mental healthcare professionals. Most interventions were educational but examined attitudinal outcomes which were generally reported to have improved following the intervention. Some studies also reported improved knowledge, behavioural intentions and/or clinical competence. When follow-up assessments were conducted these generally indicated that the positive changes had been sustained (e.g. [44, 45]), however, only a few studies had examined this.

Two studies had examined internet-based stigma reduction in relation to mental illness in general. In the first, significantly lowered scores for social distance were reported amongst Turkish psychiatrists randomly assigned to receive an instructional email about stigma, compared to controls who received a questionnaire on social distance [46]. However, this study did not include any baseline measures; a major methodological weakness. The other intervention comprised internet-based education on mental illness to professionals working in long-term care facilities in the USA, following which significant positive differences were found for all outcomes including measures of knowledge, attitudes (stereotype endorsement), empathy, self-efficacy and intended behaviour [47].

Face to face stigma reduction training for healthcare providers was conducted within Canada’s Opening Minds anti-stigma programme [48]. Thirty-seven contact-based education programmes were evaluated using a mixed methods approach, to identify the key ingredients associated with attitude change [42]; no behavioural measures were used. Table 1 provides a summary of these findings; multiple forms of contact and an emphasis on recovery were identified as the most critical ingredients.

**Table 1:** Key ingredients of anti-stigma programmes for healthcare providers

Anti-stigma ingredient
Social contact in the form of a personal testimony from a trained speaker who has lived experience of mental illness
Multiple forms or points of social contact (for example, a presentation from a live speaker and a video presentation, multiple first-voice speakers, multiple points of social contact between program participants, and people with lived experience of mental illness)
Focus on behaviour change by teaching skills that help health care providers know what to say and what to do
Engage in myth-busting
Enthusiastic facilitator or instructor who models a person-centred approach (that is, a person-first perspective as opposed to a pathology-first perspective) to set the tone and guide programme messaging
Emphasise and demonstrate recovery as a key part of its messaging

Based on findings reported on p. S21–22 in [42]

### Police officers

Criminal justice professionals are another key group for stigma reduction interventions [43]. The deinstitutionalisation of mental health services has led to a significant increase in contact between the police and those with mental illness, and it has been argued that police officers should be provided education and training to improve the outcomes of their interaction with people with mental illness. Few studies have been conducted in this area, but evidence from two is summarised below.

Pinfold and colleagues [49] evaluated a training intervention with one police force in England. Police officers’ (*n* = 109) knowledge, attitudes and behavioural intentions in relation to mental illness were assessed before and after attending two educational workshops delivered by service users, carers and people working in the field of mental health. The results indicated no changes in perceived knowledge. Also, although the intervention produced some improvements in reported attitudes, the stereotype linking people with mental health problems with violent behaviour was not successfully challenged. However, a third of the participants reported positive impacts on police work at follow-up, particularly improvements in communication between officers and persons with mental health problems.

Hansson and colleagues [50] examined the effectiveness of an anti-stigma intervention in a police officer training programme in Sweden (*n* = 120), through a controlled pre-post intervention study design with a comparison group and 6-month follow-up of the intervention group. They found that the intervention improved police officers’ attitudes, mental health literacy and intentional behaviours. These changes were generally still present 6 months later. The intervention was well received amongst the trainees, and some of its key elements have been retained in the regional police training programme.

### Students

A systematic review of the overall effect of variety of interventions delivered to student groups [51] identified 35 studies (involving 4257 students) covering a range of interventions including contact with a person with mental health problems, and education via text, lecture, film or role play. Narrative synthesis indicated that live or video-based contact with people with mental health problems were the most effective interventions in improving attitudes and reducing desire for social distance. Evidence from one study suggested that providing treatment information might enhance students’ attitudes towards the use of services [52].

## Research on anti-stigma interventions: the state of the art

Several reviews using systematic search strategies and either meta-analytic or narrative synthesis together provide a valuable summary of both the evidence for anti-stigma interventions and the state of the art in terms of evaluation methods. Reviewers have variously chosen to categorise interventions based on the anti-stigma strategy [24]; medium used [53]; type of stigma and mental disorder [54]; and length of follow-up, outcomes measured and setting [55]. While the scope of these reviews leads to extensive overlap in the papers included, this variety of categorisations makes their results difficult to compare. The various terms used for contact which is other than direct face to face contact obscures their broad similarity as forms of parasocial contact [56]. We therefore use this term in Table 2, which summarises the evidence from systematic reviews and, where not covered by systematic reviews, individual studies.

**Table 2:** Summary of evidence on interventions to reduce stigma

Type of intervention or nonexperimental exposure	Knowledge and attitudes	Behaviour
Short term face to face contact	+ SR	− SR
Short term parasocial contact	+ SR	− SR
Long term face to face contact^a^	+ IS [37, 38]	− SR
Long-term parasocial contact (with or without other interventions)	+ IS (see section on “Anti-stigma interventions for the general public”)	+ IS [39]
Education	+ SR	− SR
Protest	− SR	− SR
Structural approaches	− SR	− SR

Key: + positive evidence; − evidence lacking; SR evidence from one or more systematic reviews; IS evidence from one or more individual studies [study references in brackets]

^a^Long-term contact through knowing someone with a mental illness

A narrative review [57], conducted at the same time as one of the aforementioned systematic reviews [55] but using a broader search strategy, summarised what is known about evaluation methods for anti-stigma interventions. It was noted that studies are heterogeneous and commonly have methodological limitations (e.g. weak study designs, small samples); Low and Middle Income Country data is notably missing; and long-term follow-up data and insights on how improvements can be sustained are needed, as are studies considering service-user perspectives and discrimination and behaviour change. Last, many national programmes target both the general public and specific target groups without control groups, making it difficult to disentangle the effects of samples in target groups who may receive both interventions.

Overall, anti-stigma interventions appear to result in small-to-moderate-sized effects (as assessed using Cohen’s interpretation; [58]), and strategies based on direct or parasocial contact. At the population level, evidence points towards anti-stigma interventions resulting in improved attitudes, at least in the short term. There is also some evidence for improvements in knowledge. For anti-stigma efforts amongst specific target groups, interventions based on contact likewise seem to result in short-term benefits in terms of improved attitudes, but there is less evidence for achieving changes in knowledge.

## Directions for future interventions and research

Intervening to reduce stigma and discrimination requires a long-term, sustained commitment [59]. However, the need to improve the evaluation methods and the interventions themselves is urgent.

While contact-based education has become the most popular method for work with target groups, the evidence for its long-term impact is limited [55] and there is evidence that education is relatively more effective for youth [24]. To try to increase the effectiveness of both direct and parasocial contact, closer attention could be paid to existing literature on intergroup contact when designing and evaluating such interventions. For example, Knaak and colleagues identified six ingredients for contact-based education with health professionals [42], without relating these to the ingredients previously identified by intergroup contact theory. In contrast, another study [60] examined changing negative attitudes amongst students towards a confederate classified as a ‘former mental patient’, with an approach explicitly informed by Allport’s theory on intergroup attitudes and contact [61]. Allport’s ‘contact hypothesis’ proposes that for contact between members of in- and out-groups to lead to favourable outcomes for the out-group member, the two groups need to be afforded equal status during the interaction, and the interaction needs to involve a mutual goal. The study followed these principles, and incorporated other conditions identified in existing literature as necessary for successful intergroup contact: the opportunity to get to know the out-group member during the interaction; interaction disconfirming negative stereotype; active co-operation; and interaction including a guiding structure. After co-operative contact activities, initially prejudiced students not only described the ‘mental patient’ confederate more positively, but these improvements also generalised beyond this specific individual to the out-group overall.

Intergroup contact researchers in the UK [62] have discussed the application of intergroup contact theory to mental health-related stigma. This work examined the influence of different types of imagined contact with people with schizophrenia, and concluded that imagined contact might in in this case increase intergroup anxiety (and thus desire for social distance) unless it was purposefully structured to reflect a positive imagined contact experience. When designing anti-stigma interventions, it is therefore important to consider what factors mediate the effectiveness of intergroup contact-based strategies. A meta-analysis of over 500 studies [63] confirmed that contact can diminish prejudice through resulting in reduced anxiety about contact, and increased empathy and perspective taking. A further key factor was enhanced knowledge regarding the outgroup, however, the mediational value of this influence was weaker than that of reduced anxiety and increased empathy. More recently, the importance of threats, both realistic and symbolic, perceived by one group about the other have been identified as important mediators [56]. Realistic threats include that of violence or loss of access to resources as a result of increased contact with another group; symbolic threats are those to the values and beliefs of a group.

These mediators should be considered when designing interventions for target groups. Anxiety and perceived realistic threat are likely to be high in groups with little prior contact or knowledge, such as the general population and in particular young people. These were found to be important mediators in a study of the relationship between contact and desired avoidance among students [64]. On the other hand, groups with frequent contact are unlikely to have high levels of anxiety. Studies of stigma among health professionals have been suggested to show that clinical contact may not reduce stigma, however the circumstances of the contact have rarely been examined in relationship to intergroup contact theory. Equal status is not typical in clinical encounters with health professionals but is important for contact to succeed [65]. Intergroup contact theory likewise highlights the importance of stereotype disconfirmation and acquaintanceship; aspects which may be harder to achieve when mental health professionals see only those most severely affected by mental illness and at the times when they are most ill, and over short periods of time. Loss of empathy, for example due to short term stress or longer term burnout [66], may be a relatively more important mediator in this and other groups with contact under circumstances which are not ideal, such as emergency services personnel. Further, the type of knowledge which is effective may vary depending on the target group. Biological knowledge has been suggested as potentially having a positive effect on doctors’ attitudes [67], while a meta-regression of public attitudes surveys suggested that agreement with biological causal explanations of mental illness among the general population may have a negative effect [68].

A warning note to programmes planning or delivering contact-based interventions regards a possible unintended consequence, namely lack of progress in addressing structural discrimination. Anti-stigma programmes may try to target structural level discrimination through inclusion of target groups such as employers or health professionals. However, governmental funders of anti-stigma programmes may prefer to pay for contact and educational interventions with the aim of reducing interpersonal stigma rather than face the possible financial implications of addressing structural discrimination; likewise, stakeholders such as employers may prefer to receive interpersonal stigma interventions. While providing contact-based education may be effective in improving the experience of people with mental health problems either as employees or service users, there is a danger that this results in reduced attention and effort in relation to structural change [56]. Measuring structural discrimination is complex but a number of approaches have been recently reviewed along with the evidence for its impact and the effects of addressing it [69].

The effectiveness of intergroup contact is exploited by anti-stigma programmes in several ways. For those with no familiarity, they provide parasocial contact. They may also enhance the effectiveness of pre-existing face to face contact through familiarity with a friend, relative or colleague; raising awareness or increasing mental health literacy may cause people to realise they know someone with a mental health problem, and the existence of such campaigns reflects institutional support for this contact, an important ingredient for successful contact [61]. Strategies such as Time to Change’s ‘Time to Talk Day’ (http://www.time-to-change.org.uk/timetotalkday) may encourage extended contact, that is, the effect of having a friend who knows someone with a mental illness. Extended contact has been shown to be associated with lower prejudice regarding other groups [56]. However, the influence of extended contact also has potentially negative consequences. Selective description of people with mental illnesses or of events involving them such as by health professionals or emergency services personnel may act to confirm stereotypes, for example anecdotes of violent incidents or severe disability. On the other hand, these professionals are also potential anti-stigma agents through extended contact should they give others a more balanced view of their work. To our knowledge, this potential has not so far been exploited by anti-stigma programmes.

We identify three further means to maximise the effectiveness of interventions for target groups. First, preparatory work can increase the understanding of the target group and the context of their contact with the stigmatised group [70], for example using qualitative interviews or ethnography to inform intervention design. This can inform, for example, the choice of contact type between face to face versus parasocial contact, which is more appropriate in settings in which disclosure poses risks for people with mental illness and/or their family members. These methods could also elucidate the influences of structural and organisational level discrimination on individual level interactions and the experience of people with mental health problems [32, 71], allowing the identification of structural level interventions. Second, it should be noted that in both England and Canada, there were gender-based differences in responses to the anti-stigma programmes, with men reporting less contact with people with a mental illness than women. As such contact is important for improved stigma reduction outcomes, future efforts might benefit from gender-based approaches [30]. Last, those with pedagogical expertise could be involved to ensure the use of evidence-based teaching and assessment methods when conducting skills training.

Regarding evaluation methods, our general design recommendations include: randomised designs for interventions tailored to specific groups; improved reporting of study procedures; validated and appropriate outcome measures which match the intervention content, including measures of behaviour and impact on people with mental health problems; better controlling for confounding factors and potential social desirability bias [72]; increased sample sizes; better sampling procedures to increase representativeness, and follow-up data collection beyond the immediate end of the intervention. Process evaluation to assess the context, implementation and mechanisms of action [73] of interventions would facilitate the delivery of more effective interventions in future. An example is the process model developed by Knaak and colleagues [74]. As it seems that short-term interventions often only have a short-term impact, the implication is that we need to study longer term interventions and to use the interim process and outcome data to improve the interventions along the way.

Evidence on the long-term impact of longer term interventions such as national anti-stigma programmes using mass media is hard to obtain; however, it is hard to show a causal association in these circumstances. While it is not usually possible to conduct randomised studies of population-based interventions, other quasi-experimental designs [75] should be considered to examine the extent to which change is attributable to the campaign rather than to secular changes. Otherwise, the use of repeated surveys can be used not just to assess changes in outcomes over time, but to examine whether the outcomes are associated with campaign awareness on the part of the target group [76, 77].

The many directions which could be taken by anti-stigma interventions and their evaluation make this an exciting and challenging field. For those working in this field, there is a balance to strike if we are to better identify effective and replicable interventions. On one hand, we must acknowledge that the goals of those who deliver programmes and those who evaluate them overlap only partially; while evaluators want to find positive results they must work independently so that their results are credible and informative. On the other, collaboration is essential if we are to realise the potential of developing and applying theory-based interventions and evaluation designs.
